# Lauric Acid, a Dietary Saturated Medium-Chain Fatty Acid, Elicits Calcium-Dependent Eryptosis

**DOI:** 10.3390/cells10123388

**Published:** 2021-12-01

**Authors:** Mohammad A. Alfhili, Ghadeer S. Aljuraiban

**Affiliations:** 1 Chair of Medical and Molecular Genetics Research, Department of Clinical Laboratory Sciences, College of Applied Medical Sciences, King Saud University, Riyadh 12372, Saudi Arabia; 2 Department of Community Health Sciences, King Saud University, Riyadh 12372, Saudi Arabia; galjuraiban@ksu.edu.sa

**Keywords:** lauric acid, eryptosis, hemolysis, calcium, cardiovascular disease

## Abstract

Cardiovascular diseases (CVD) are a leading cause of mortality worldwide, and dietary habits represent a major risk factor for dyslipidemia; a hallmark of CVD. Saturated fatty acids contribute to CVD by aggravating dyslipidemia, and, in particular, lauric acid (LA) raises circulating cholesterol levels. The role of red blood cells (RBCs) in CVD is increasingly being appreciated, and eryptosis has recently been identified as a novel mechanism in CVD. However, the effect of LA on RBC physiology has not been thoroughly investigated. RBCs were isolated from heparin-anticoagulated whole blood (WB) and exposed to 50–250 μM of LA for 24 h at 37 °C. Hemoglobin was photometrically examined as an indicator of hemolysis, whereas eryptosis was assessed by Annexin V-FITC for phosphatidylserine (PS) exposure, Fluo4/AM for Ca^2+^, light scatter for cellular morphology, H_2_DCFDA for oxidative stress, and BODIPY 581/591 C11 for lipid peroxidation. WB was also examined for RBC, leukocyte, and platelet viability and indices. LA caused dose-responsive hemolysis, and Ca^2+^-dependent PS exposure, elevated erythrocyte sedimentation rate (ESR), cytosolic Ca^2+^ overload, cell shrinkage and granularity, oxidative stress, accumulation of lipid peroxides, and stimulation of casein kinase 1α (CK1α). In WB, LA disrupted leukocyte distribution with elevated neutrophil-lymphocyte ratio (NLR) due to selective toxicity to lymphocytes. In conclusion, this report provides the first evidence of the pro-eryptotic potential of LA and associated mechanisms, which informs dietary interventions aimed at CVD prevention and management.

## 1. Introduction

Cardiovascular diseases (CVD) are a leading cause of mortality and disability worldwide [1]. In fact, the number of cases of CVD doubled over the past three decades to 523 million cases in 2019, contributing to 18.6 million deaths that same year [1]. These figures correspond to 34.4 million years lived with disability [1]. To address this issue, implementing strategies to deal with underlying contributors to CVD, such as hypertension, hyperglycemia, inflammation, obesity, and dyslipidemia, can have major health benefits [1].

Dietary intakes are a major risk factor for these underlying conditions [1,2]. Guidelines aimed at increasing fruit, vegetable, nut, and legume consumption and reducing intakes of refined carbohydrates, sodium, and saturated fats have been recommended to address the risk of CVD [3]. In general, the basis for guidelines suggesting reduced intakes of saturated fatty acids provides evidence of their contribution to dyslipidemia as fatty acids (FAs) in the diet are hydrolyzed into free fatty acids (FFAs) [4]; circulating in the bloodstream and acting as an energy source for organs and regulating cellular function [5], including lymphocyte proliferation [6], activation by antigens [7], and stimulation of cell death [8,9], among others [10]. It is suspected that FFAs contribute to the risk of non-communicable diseases, for instance, higher levels of FFAs have been associated with sudden cardiac death [11], heart failure [12], and diabetes [13].

While recommendations to limit intakes of saturated fats are widespread, these recommendations fail to consider the varying health effects of different saturated FAs in the diet [14,15]. Lauric acid (LA; C12:0) is a medium-chain saturated FA and a major component of tropical oils such as coconut and palm oils [16] that has been demonstrated to have the largest cholesterol-raising effect of all FAs, raising low-density lipoprotein-cholesterol (LDL-C) and high-density lipoprotein-cholesterol (HDL-C) [17]. As a result, LA increases total cholesterol levels and leads to improvements in the total cholesterol to HDL-C ratio [17,18], which is important for estimating CVD risk, with higher HDL cholesterol-lowering the risk of CVD [19]. Therefore, the cardiovascular impact of LA in terms of dyslipidemia may be benign rather than detrimental.

Oils rich in LA, however, may still be harmful in terms of CVD through other mechanisms [17]. The composition of the food in which FAs are found can contribute to the impact of FAs on health [14,15,20]. Of relevance, many of the studies describing the cardiovascular impacts of LA have used LA-rich fats or oils that are mainly comprised of LA, but also other FAs, such as palmitic and myristic acids [20,21,22,23,24,25], shown to have differential health impacts [17,26]. Two recent studies have compared the effect of isolated LA on inflammation and reported that it reduced inflammation in mouse models [27] and human primary myotubes [26] to a greater extent than palmitic acid, emphasizing the importance of separating FAs to examine their unique impact. Studies investigating the effect of purified or isolated LA to understand its specific effects on cardiometabolic processes and potential use in prevention and treatments are needed [20].

Cell membranes are a complex amalgam of proteins, lipids, and carbohydrates [28]. While proteins maintain the architectural integrity of membranes and mediate substance trafficking, lipids, namely phospholipids and sterols, preserve membrane fluidity and participate in signal transduction. Despite the continuous movement of proteins and lipids within the membrane, the outer and inner leaflets significantly differ with regard to their lipid composition. Whereas phosphatidylcholine (PC) and sphingolipids dominate the exterior side of the membrane, phosphatidylserine (PS) and phosphatidylethanolamine (PE) are confined to the interior of the cell [28]. However, when a cell undergoes apoptosis, loss of membrane asymmetry culminates in PS externalization, which serves to impart a negative charge to the membrane that primes the cell for phagocytic engulfment [29].

Hydrophobic and amphipathic compounds have long been known to exhibit a dual interaction with the cell membrane. Earlier studies on red blood cells (RBCs; erythrocytes) revealed that anesthetics cause hemolysis in isotonic solutions but prevent hypotonic lysis [30]. This is attributed to the intercalation of the hemolytic agent and subsequent membrane expansion or ion efflux under hypotonic conditions [31,32], which increases the hemolytic threshold of the cell. In particular, FFAs exhibit a similar biphasic interaction with RBCs [33]. Nonetheless, many of the underlying molecular mechanisms remain largely unknown. Furthermore, while it is known that oxidative stress plays an important role in the progression of atherosclerosis via the oxidation of LDL-C particles [34,35,36], the contribution of oxidative stress to other mechanisms, such as hemolysis and eryptosis, has not been fully investigated.

In light of the growing appreciation of the role of RBCs, hemolysis, and eryptosis in CVD, this study was initiated to investigate the cytotoxic potential of dietary medium-chain fatty acid, LA, in human RBCs, and to identify the underlying molecular mechanisms.

## 2. Materials and Methods

### 2.1. Blood Collection and RBC Purification

This study was approved by the Ethics Committee/IRB of the College of Medicine, King Saud University (Project No. E-20-4544) and conducted according to the Declaration of Helsinki. Heparinized blood samples were collected from 12 healthy volunteers and RBCs were isolated by centrifugation at 3000 rpm for 20 min and repeated washing in phosphate-buffered saline (PBS) [37]. For complete blood count analysis, K_2_-EDTA-anticoagulated whole blood (WB) was used [38].

### 2.2. Chemicals and Reagents

LA (CAS: 143-07-7), palmitic acid (PA; CAS: 57-10-3), Annexin-V-FITC, Fluo4/AM, 2’,7’-dichlorodihydrofluorescein diacetate (H_2_DCFDA), BODIPY 581/591 C11, calcium ionophore ionomycin (IMC), pan-caspase inhibitor zVAD(OMe)-FMK (zVAD), p38 MAPK inhibitor SB203580, casein kinase 1α (CK1α) inhibitor D4476, reduced glutathione (GSH), cyclooxygenase (COX) inhibitor aspirin (Asp), glucose transporter 1 (Glut1) inhibitor WZB117, and erythropoietin (EPO) were obtained from Solarbio Life Science (Beijing, China), whereas Amplex^TM^ Red Reagent (10-acetyl-3,7-dihydrophenoxazine) was obtained from Thermo Fisher Scientific (Waltham, MA, USA). LA and PA were made as 100 mM stock solutions in dimethylsulfoxide (DMSO) and stored at −80 °C. Ringer solutions were prepared essentially as described elsewhere [37], containing 1 mM of CaCl_2_ when present.

### 2.3. Hemolysis

Control and experimental cells were pelleted by centrifugation at 13,300× *g* for 1 min at 20 °C and the supernatant was assayed for hemoglobin content by measuring light absorbance at 405 nm using xMark^™^ microplate spectrophotometer (Bio-Rad Laboratories, Hercules, CA, USA) [39]. Cells suspended in distilled water represented 100% hemolysis and LA-induced hemolysis was derived as a percentage relative to total hemolysis as follows:

(1) $$\%\;\mathrm{Hemolysis}=\frac{\mathrm{LA}-\mathrm{induced}\;\mathrm{hemoglobin}\;\mathrm{release}}{\mathrm{water}-\mathrm{induced}\;\mathrm{hemoglobin}\;\mathrm{release}}\times 100$$

### 2.4. Phosphatidylserine Exposure

Cells were stained with 1% Annexin V-FITC and examined with CytoFLEX™ flow cytometer (Beckman Coulter, Brea, CA, USA) as previously reported [39]. A total of 50 μL of control and experimental cells were washed in PBS, suspended in 150 μL of 1% Annexin V-FITC solution containing 5 mM of CaCl_2_ for 15 min at room temperature away from light and then examined at an excitation light of 488 nm and emission light of 512 nm.

### 2.5. Erythrocyte Sedimentation Rate (ESR)

Control and experimental cells in WB were allowed to vertically sediment in Westergren tubes at room temperature for 60 min away from light, and the distance traveled in millimeters was then recorded [40].

### 2.6. Intracellular Calcium

Cytosolic calcium was detected with 2.5 μM of Fluo4/AM as per standard protocols [41].

### 2.7. Cellular Morphology

Forward scatter (FSC) and side scatter (SSC) of light were measured by flow cytometry as surrogates for cell size and surface granularity, respectively [37].

### 2.8. Oxidative Stress

Total reactive oxygen species (ROS) were labeled with 5 μM of H_2_DCFDA as previously reported [42]. Control and experimental cells were washed in PBS, suspended in Ringer buffer with 1 mM of CaCl_2_ and 5 μM of H_2_DCFDA for 30 min at 37 °C away from light. DCF was excited by the blue laser at 488 nm and the green fluorescence was detected at 512 nm.

### 2.9. Hydrogen Peroxides (H_2_O_2_) and Superoxide Anions (SOA)

The generation rate of H_2_O_2_ was determined by Amplex^TM^ Red as previously reported [43], while SOA formation was estimated by the reduction of ferricytochrome C as detailed in [44].

### 2.10. Antioxidant Enzymes

Catalase (CAT), superoxide dismutase (SOD), and glutathione peroxidase (GPx) activities were measured using colorimetric kits (Solarbio Life Science) as previously described [45].

### 2.11. Glutathione Status

Detection of 2-nitro-5-mercaptobenzoic acid from Ellman’s reagent (5,5’-dithiobis-(2-nitrobenzoic acid); DTNB) was used as a surrogate for GSH and oxidized glutathione (GSSG) content as documented elsewhere [46].

### 2.12. Lipid Peroxidation

Homogeneous 50 μL aliquots of control and LA-treated cells were washed in Ringer solution, resuspended in 150 μL of 5 μM of BODIPY 581/591 C11 staining solution, and incubated at 37 °C in the dark for 30 min. Cells were then washed twice, excited by 488-nm blue laser, and fluorescence of emitted light was captured at 530 nm [47,48].

### 2.13. Protein Carbonylation (PCC)

Oxidized protein content was determined following derivatization by 2,4-dinitrophenylhydrazine (DNPH) as per [49].

### 2.14. Complete Blood Count

Control and 100 μM LA-treated WB were examined for reticulocyte, RBC, white blood cell (WBC), and platelet viability and indices on a Sysmex XN Series hematology analyzer (Kobe, Hyogo, Japan), as previously described [38].

### 2.15. Statistical Analysis

Data are represented as means ± standard error of the mean (SEM) of at least nine determinations from three independent experiments (*n* = 9–18). Flow cytometry data as arbitrary units (a.u.) or percentages were analyzed by FlowJo^™^ Software v10.7.2 (Becton, Dickinson and Company, Ashland, OR, USA), and GraphPad Prism v9.2.0 (GraphPad Software, Inc., San Diego, CA, USA) was used for statistical analysis. Two groups were analyzed by unpaired, two-tailed Student t-test, whereas multiple means were analyzed by one-way ANOVA and either Dunnett’s or Tukey’s post-hoc test, or by two-way ANOVA followed by Šidák correction. In all cases, a cutoff *p*-value of <0.05 was set for statistical significance. Asterisks * (*p* < 0.05), ** (*p* < 0.01), *** (*p* < 0.001), and **** (*p* < 0.0001) indicate significantly different values from control conditions whereas ^#^ (*p* < 0.05), ^##^ (*p* < 0.01), and ^###^ (*p* < 0.001) indicate statistical significance compared to LA-treated cells.

## 3. Results

### 3.1. LA stimulates Calcium-Independent Hemolysis

Hemolysis exacerbates oxidative injury associated with CVD. To assess the hemolytic potential of LA, RBCs were treated with vehicle control (0.025% DMSO) or with 50, 100, and 250 μM of LA for 24 h at 37 °C, and hemolysis was then measured.

**Figure 1:**
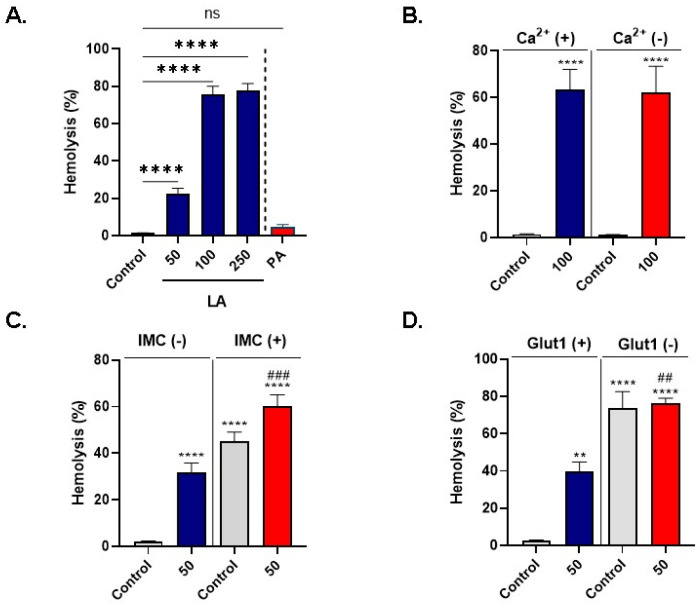
LA stimulates Ca^2+^-independent hemolysis. (**A**) LA but not PA induces dose-dependent hemolysis. (**B**) Ca^2+^ sensitivity of LA-induced hemolysis. (**C**) Ionophoric challenge with IMC does not aggravate LA-induced hemolysis. (**D**) Inhibition of glucose uptake by Glut1 inhibitor WZB117 does not exasperate LA-induced hemolysis. ns indicates not significant, ** (*p* < 0.01) and **** (*p* < 0.0001) indicate significantly different values from control conditions whereas ^##^ (*p* < 0.01) and ^###^ (*p* < 0.001) indicate statistical significance compared to LA-treated cells.

As shown in Figure 1A, the rate of hemolysis increased from 1.34 ± 0.08% (control) to 22.60 ± 2.80% (50 μM), 75.63 ± 4.43% (100 μM), and 77.91 ± 3.60% (250 μM). In comparison, no signifciant hemolysis was observed at 250 μM of PA.

Next, we examined the role of extracellular Ca^2+^ in LA-mediated hemolysis by incubating control and experimental cells in standard and Ca^2+^-free Ringer solutions. Figure 1B shows that no statistically significant difference was observed following treatment with 100 μM of LA in presence or absence of extracellular Ca^2+^ (63.49 ± 8.44% vs. 62.12 ± 11.27%). Moreover, we investigated the possible interaction of LA with IMC by exposing the cells to 50 μM of LA with and without 0.25 μM of IMC for 24 h at 37 °C (dose-dependence hemolysis of IMC is shown in Figure S1). As depicted in Figure 1C, compared to control values (2.05 ± 0.29%), significant hemolysis was detected following isolated treatment with either LA (31.92 ± 3.92%) or IMC (45.09 ± 4.03%), while cells co-treated with both LA and IMC exhibited a significantly higher hemolytic rate (60.01 ± 5.07%) compared to treatment with LA alone.

Finally, since glucose is the major source of energy for RBCs [50], and the lack of which sensitizes the cells to hemolysis [51], we probed the involvement of Glut1 in LA-induced hemolysis by incubating the cells with 50 μM of LA in the presence and absence of 50 μM of WZB117 for 24 h at 37 °C. Figure 1D shows that both LA (39.75 ± 5.0%) and WZB117 (73.80 ± 8.79) caused significant hemolysis, whereas the combined treatment with both resulted in significantly more pronounced hemolysis than LA alone (76.32 ± 2.76).

Altogether, these data indicate that LA exerts a dose-responsive and Ca^2+^-independent hemolysis, an effect unrelated to ionophoric challenge or inhibition of Glut1 activity.

### 3.2. LA Elicits Calcium-Dependent Eryptosis

**Figure 2:**
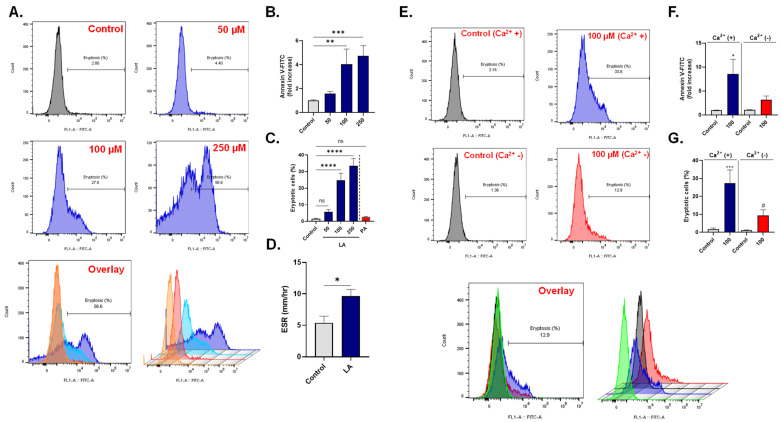
LA elicits Ca^2+^-dependent PS exposure. (**A**) Representative histograms of Annexin V-FITC of cells exposed to 0–250 μM of LA. (**B**) LA-induced fold increase in mean intensity fluorescence (MFI) of Annexin V-FITC. (**C**) LA but not PA induces an increase in the percentage of Annexin V-positive cells. (**D**) LA-induced increase in ESR. (**E**) Representative histograms of Annexin V-FITC of cells exposed to 0–100 μM of LA with and without Ca^2+^. (**F**) LA-induced fold increase in MFI of Annexin V-FITC with and without Ca^2+^. (**G**) LA-induced fold increase in the percentage of Annexin V-positive cells with and without Ca^2+^. ns not significant, * (*p* < 0.05), ** (*p* < 0.01), *** (*p* < 0.001), and **** (*p* < 0.0001) indicate significantly different values from control conditions whereas ^#^ (*p* < 0.05) indicate statistical significance compared to LA-treated cells.

Eryptosis is a recognized contributor to CVD [52]. In order to examine the eryptotic potential of LA, cells were incubated with 50–250 μM of LA for 24 h at 37 °C and PS exposure was then detected. The results in Figure 2A,B indicate that LA significantly increases Annexin V-FITC fluorescence from control values of 1.0 ± 0.07 folds to 4.04 ± 1.26 folds (100 μM) and 4.74 ± 0.85 folds (250 μM). Congruently, as seen in Figure 2C, the percentage of eryptotic cells was significantly elevated from 1.64 ± 0.26% in the case of control to 24.79 ± 4.14% (100 μM) and 33.53 ± 4.27% (250 μM). Again, no significant eryptosis was observed at 250 μM of PA.

Since eryptosis leads to enhanced adherence of RBCs to the endothelium [47], we were prompted to assess the influence of LA on the ESR. Figure 2D shows that the ESR significantly increased from 5.37 ± 1.08 mm/h in control cells to 9.62 ± 1.06 mm/h.

Next, the importance of Ca^2+^ in LA-induced eryptosis was examined. Our results in Figure 2E,F show that removal of extracellular Ca^2+^ significantly diminished LA-induced Annexin V-FITC fluorescence (8.53 ± 3.08 folds vs. 3.19 ± 0.76 folds). Likewise, the percentage of eryptotic cells significantly decreased from 27.37 ± 7.41% in the presence of Ca^2+^ to 9.44 ± 3.13% upon elimination of Ca^2+^ (Figure 2G).

Accordingly, LA is a novel stimulator of Ca^2+^-dependent eryptosis, which is associated with increased ESR.

### 3.3. LA promotes Extracellular Calcium Influx

**Figure 3:**
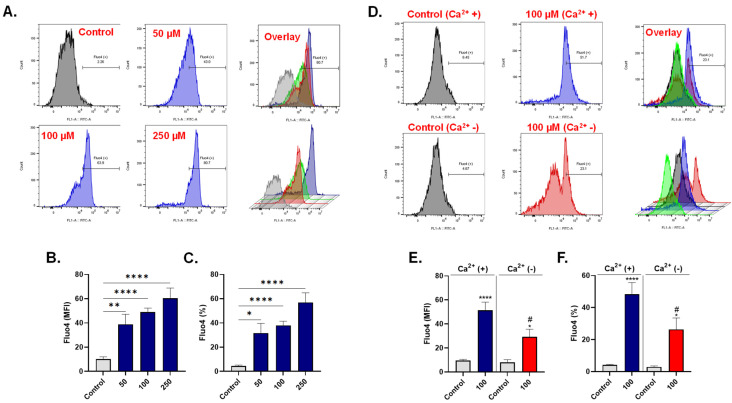
LA promotes extracellular Ca^2+^ influx. (**A**) Representative histograms of Fluo4 of cells exposed to 0–250 μM of LA. (**B**) LA-induced increase in MFI of Fluo4. (**C**) LA-induced increase in the percentage of Fluo4-positive cells. (**D**) Representative histograms of Fluo4 of cells exposed to 0–100 μM of LA with and without Ca^2+^. (**E**) LA-induced increase in Fluo4 MFI with and without Ca^2+^. (**F**) LA-induced increase in the percentage of Fluo4-positive cells with and without Ca^2+^. ns indicates not significant, * (*p* < 0.05), ** (*p* < 0.01), and **** (*p* < 0.0001) indicate significantly different values from control conditions whereas ^#^ (*p* < 0.05) indicate statistical significance compared to LA-treated cells.

Eryptosis is triggered by disturbances in Ca^2+^ homeostasis. To measure cytosolic Ca^2+^, we labeled control and LA-exposed cells with intracellular Ca^2+^ indicator Fluo4/AM. As Figure 3A,B shows, LA caused a significant increase in Fluo4 fluorescence of control cells from 10.23 ± 1.80 a.u. to 38.93 ± 8.25 a.u. (50 μM), 48.92 ± 3.40 a.u. (100 μM), and 60.49 ± 8.36 a.u. (250 μM). Similarly, Figure 3C shows a significant increase in the percentage of cells with accumulated Ca^2+^ from control values of 4.42 ± 0.70% to 31.64 ± 8.01% (50 μM), 38.06 ± 3.39% (100 μM), and 56.90 ± 7.94% (250 μM).

Furthermore, we investigated whether the observed increase in Ca^2+^ was secondary to the influx of extracellular Ca^2+^. Figure 3D,E indicate that cells exposed to 100 μM of LA in the absence of Ca^2+^ exhibited significantly reduced Fluo4 fluorescence (29.26 ± 6.38) in comparison to those exposed to 100 μM of LA in the presence of Ca^2+^ (51.51 ± 6.73 a.u.). This was also reciprocated when the percentage of LA-treated cells with increased Ca^2+^ was examined with (48.34 ± 7.26%) and without (26.43 ± 7.07%) extracellular Ca^2+^ (Figure 3F).

Collectively, these data suggest that LA-induced eryptosis is associated with elevated intracellular Ca^2+^ that is mainly triggered by excessive influx from the extracellular space.

### 3.4. LA Causes Cell Shrinkage and Granularity

**Figure 4:**
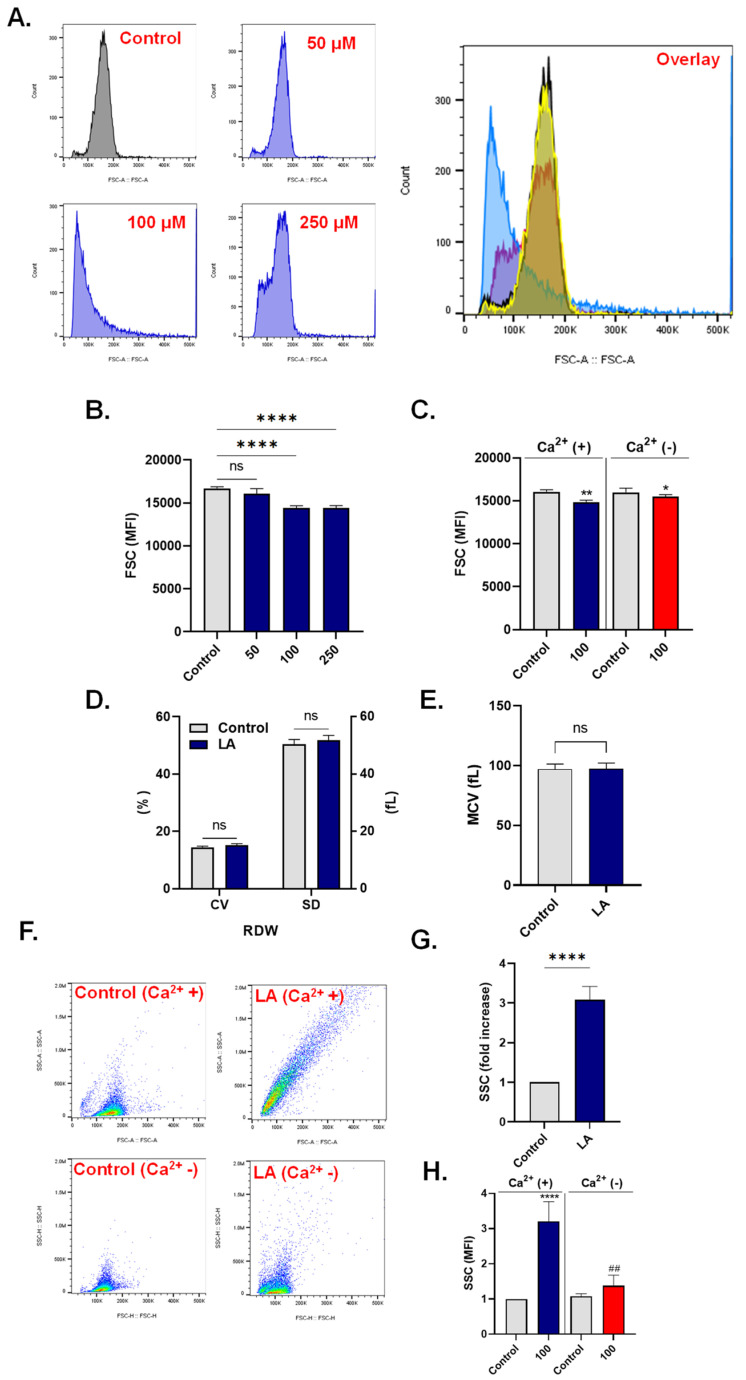
LA causes loss of cellular volume and increased granularity. (**A**) Representative histograms of FSC of cells exposed to 0–250 μM of LA. (**B**) LA-induced decrease in MFI of FSC. (**C**) LA-induced decrease in FSC MFI with and without Ca^2+^. (**D**) RDW expressed in terms of coefficient of variation (CV) and standard deviation (SD). (**E**) MCV. (**F**) Dot plot scattergrams of SSC and FSC of control and LA-treated cells in the presence and absence of Ca^2+^. (**G**) Fold increase in SSC. (**H**) Fold increase in SSC in the presence and absence of Ca^2+^. ns indicates not significant, * (*p* < 0.05), ** (*p* < 0.01), and ****(*p* < 0.0001) indicate significantly different values from control conditions whereas ^##^ (*p* < 0.01) indicates statistical significance compared to LA-treated cells.

Accumulation of Ca^2+^ is typically followed by water efflux and loss of cellular volume. Control and experimental cells were examined for their size with FSC. As depicted in Figure 4A,B, control values of 16,702 ± 197.3 a.u. significantly decreased following treatment with 100 μM of LA to 14,410 ± 258.4 a.u. and following treatment with 250 μM of LA to 14,384 ± 311.5 a.u. Again, the participation of extracellular Ca^2+^ was evaluated by measuring FSC values with and without Ca^2+^. Our results in Figure 3C show no significant difference in FSC levels in cells exposed to 100 μM of LA with (14,855 ± 226.4 a.u.) or without (15,471 ± 253.1 a.u.) Ca^2+^. Notably, testing volume changes in WB showed no significant effect of LA on either red cell distribution width (RDW; Figure 4D) or mean corpuscular volume (MCV) values (Figure 4E).

Another morphological hallmark of eryptosis is membrane blebbing and acanthocytosis. We thus investigated changes in SSC patterns following exposure to 100 μM of LA. Figure 4F,G show a significant increase in SSC values of exposed cells by 3.08 ± 0.33 folds compared to control conditions. Unlike FSC, extracellular Ca^2+^ deprivation significantly abrogated LA-induced elevation in SSC (3.20 ± 0.55 folds vs. 1.38 ± 0.29 folds) as seen in Figure 4H.

Altogether, these observations suggest that LA-induced eryptosis is accompanied by significant loss of cellular volume independently of Ca^2+^ elevation, and the formation of acanthocytes secondary to Ca^2+^ influx.

### 3.5. LA Triggers Oxidative Stress

**Figure 5:**
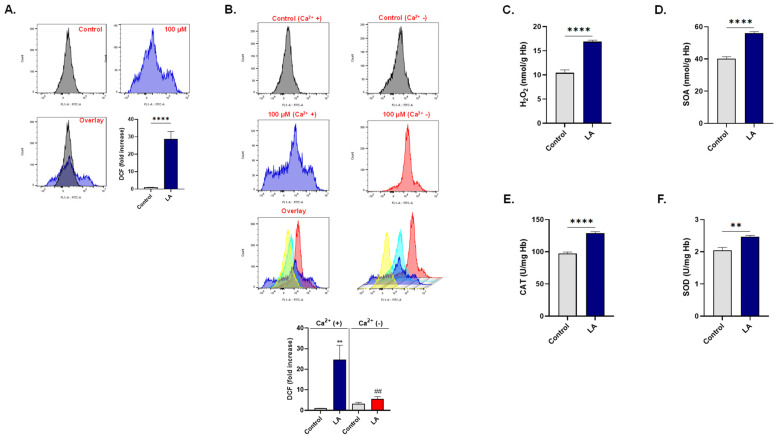
LA triggers Ca^2+^-dependent oxidative stress. (**A**) Representative histograms and fold increase in MFI of DCF fluorescence of cells exposed to 0–100 μM of LA. (**B**) Representative histograms and fold increase in MFI of DCF fluorescence of cells exposed to 0 or 100 μM of LA in presence or absence of Ca^2+^. (**C**) Intracellular content of H_2_O_2_ in cells treated with 0 or 100 μM of LA. (**D**) Intracellular content of SOA in cells treated with 0 or 100 μM of LA. (E) CAT activity in cells treated with 0 or 100 μM of LA. (**F**) SOD activity in cells treated with 0 or 100 μM of LA. ** (*p* < 0.01) and **** (*p* < 0.0001) indicate significantly different values from control conditions whereas ^##^ (*p* < 0.01) indicates statistical significance compared to LA-treated cells.

A pivotal mechanism leading up to eryptosis is oxidative stress. Cells were therefore assessed for ROS levels following exposure to 100 μM of LA. In Figure 5A, it is shown that ROS levels in treated cells are significantly elevated by 28.67 ± 4.34 folds compared to control cells.

Moreover, removal of Ca^2+^ significantly rescinded LA-induced ROS accumulation of 24.71 ± 6.94 folds to 5.58 ± 1.08 folds as depicted in Figure 5B.

As DCF is a general ROS indicator, we were prompted to identify specific free radicals responsible for the imbalanced redox status instigated by LA. As seen in Figure 5C,D, both H_2_O_2_ and SOA radicals were significantly increased in LA-treated cells from 10.44 ± 0.61 to 16.88 ± 0.30 nmol/g Hb and from 40.19 ± 1.26 to 56.18 ± 0.82 nmol/g Hb, respectively. Accordingly, the activities of antioxidant enzymes responsible for quenching these two radicals were also found to be significantly enhanced in LA-treated cells. Figure 5E shows increased CAT activity from 97.20 ± 2.37 U/mg Hb to 129.0 ± 2.24 U/mg Hb, while SOD activity in Figure 5F was elevated from 2.04 ± 0.09 to 2.46 ± 0.04 U/mg Hb.

**Figure 6:**
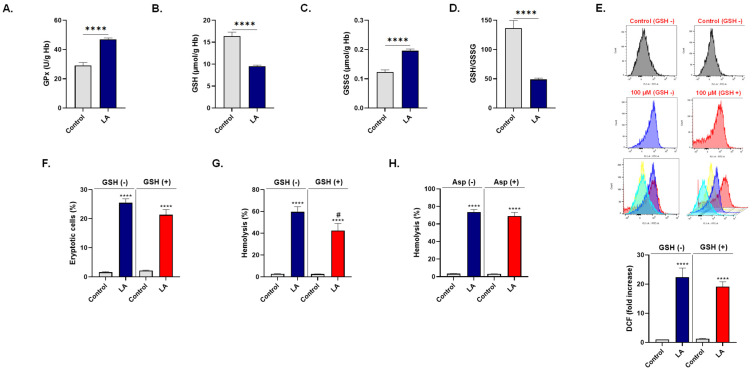
LA disrupts glutathione homeostasis. (**A**) GPx activity in control and experimental (100 μM of LA) cells. (**B**) Intracellular GSH content. (**C**) Intracellular GSSG content. (**D**) GSH/GSSG ratio. (**E**) Representative histograms and arithmetic means of fold increase in MFI of DCF fluorescence with or without 100 μM of LA in the presence or absence of 20 μM of GSH. (**F**) Eryptosis and (**G**) hemolysis of cells treated with 100 μM of LA in presence or absence of 20 μM of GSH. (**H**) Hemolysis with or without 100 μM of LA in the presence or absence of 50 μM of Asp. **** (*p* < 0.0001) indicates significantly different values from control conditions whereas ^#^ (*p* < 0.05) indicates statistical significance compared to LA-treated cells.

In light of the oxidative stress observed upon LA exposure, we sought to determine the glutathione status and the role played by this non-enzymatic antioxidant in the presence of LA. As shown in Figure 6A, LA significantly increased the activity of GPx from 29.17 ± 1.94 to 48.27 ± 1.48 U/g Hb. This was accompanied by a significant reduction in GSH (16.40 ± 0.90 to 9.52 ± 0.26 μmol/g Hb; Figure 6B) and concurrent accumulation of GSSG (0.12 ± 0.007 to 0.19 ± 0.006 μmol/g Hb; Figure 6C), thereby disturbing the GSH/GSSG ratio (136.5 ± 13.13 to 48.98 ± 2.23; Figure 6D).

Next, we examined the possible role of ROS in LA-induced eryptosis and hemolysis by supplementing the cells with 20 μM of GSH. As results in Figure 6E indicate, GSH slightly decreased LA-induced DCF fluorescence (22.42 ± 3.07 to 19.17 ± 1.60 folds), but this effect did not reach statistical significance. The percentage of eryptotic cells upon LA exposure was also decreased but with no statistical significance (25.47 ± 1.39% to 21.34 ± 1.81%; Figure 6F). Contrary to eryptosis, LA significantly increased the rate of hemolysis from 2.80 ± 0.13% to 59.55 ± 4.78% (Figure 6G), which was significantly diminished to 42.45 ± 6.49% by addition of GSH (Figure 6H).

Furthermore, given the crosstalk between oxidative stress and inflammation, we evaluated whether Asp would rescue the cells from the hemolytic effect of LA. Figure 6F shows no significant difference in hemolysis between cells exposed to 100 μM of LA without or with 50 μM of Asp (73.73 ± 2.75% vs. 69.05 ± 4.06%).

**Figure 7:**
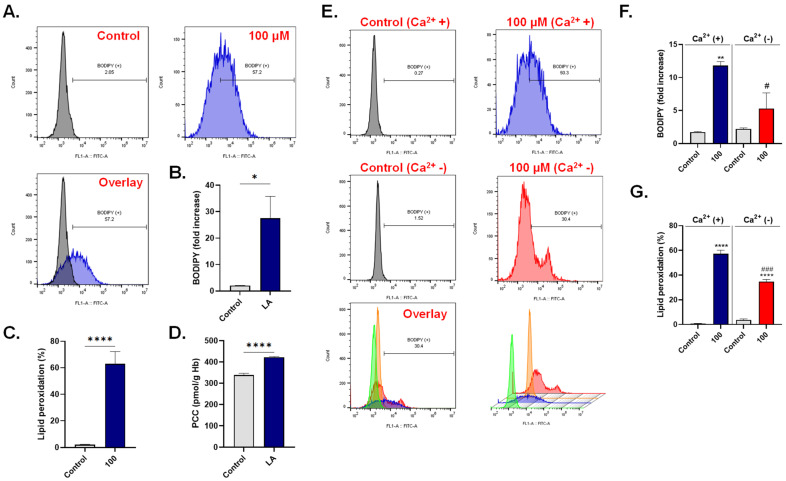
LA triggers Ca^2+^-dependent lipid peroxidation and protein carbonylation. (**A**) Representative histograms of BODIPY fluorescence of cells exposed to 0–100 μM of LA. (**B**) LA-induced fold increase in MFI of BODIPY. (**C**) LA-induced increase in the percentage of cells with high lipid peroxides. (**D**) PCC content in cells exposed to 0 or 100 μM of LA. (**E**) Representative histograms of BODIPY fluorescence of cells exposed to 0–100 μM of LA in presence or absence of Ca^2+^. (**F**) LA-induced BODIPY fold increase with and without Ca^2+^. (**G**) LA-induced increase in the percentage of cells with high lipid peroxides with and without Ca^2+^. * (*p* < 0.05), ** (*p* < 0.01), and **** (*p* < 0.0001) indicate significantly different values from control conditions whereas ^#^ (*p* < 0.05) and ^###^ (*p* < 0.001) indicate statistical significance compared to LA-treated cells.

A major consequence to ROS accumulation is peroxidative damage to lipid molecules. In Figure 7A,B, our results show discernable lipid peroxidation upon treatment with 100 μM of LA reflected as a significant increase in overall BODIPY fluorescence by 27.53 ± 8.30 folds and in the percentage of cells with lipid peroxides from 2.06 ± 0.40% to 63.02 ± 9.25% (Figure 7C).

Oxidative injury to proteins manifests as carbonylation of amino acid residues, and PCC content was thus determined and also found to be significantly increased upon treatment with 100 μM of LA (338.9 ± 8.12 to 422.8 ± 2.72 pmol/g Hb) as seen in Figure 7D. Similar to ROS, exclusion of extracellular Ca^2+^ significantly decreased LA-induced BODIPY fluorescence (11.81 + 0.61 folds to 5.32 + 2.35 folds), as shown in Figure 7E,F, and the proportion of cells with high lipid peroxides (57.20 + 3.06% to 34.80 + 1.70%) as shown in Figure 7G.

These results point to a central role for oxidative stress in LA-induced cell death.

### 3.6. LA-Induced Hemolysis Is Mitigated by D4476

In order to screen for signaling pathways involved in LA-induced cell death, hemolysis was measured following incubation of RBCs with 100 μM of LA in presence or absence of 100 μM of zVAD, 100 μM of SB203580, 20 μM of D4476, or 2 mU/mL of EPO, for 24 h at 37 °C.

**Figure 8:**
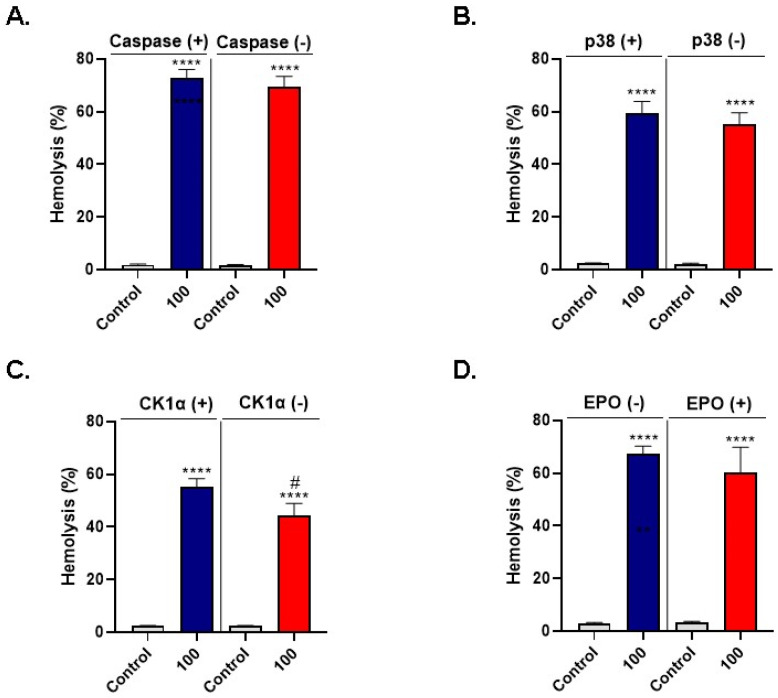
LA-induced hemolysis is mitigated by D4476. Hemolytic rates under inhibition of (**A**) caspase by zVAD, (**B**) p38 MAPK by SB203580, and (**C**) CK1α by D4476. (**D**) LA-induced hemolysis under EPO supplementation. **** (*p* < 0.0001) indicates significantly different values from control conditions whereas ^#^ (*p* < 0.05) indicates statistical significance compared to LA-treated cells.

Inhibition of caspase did not significantly ameliorate LA-induced hemolysis (72.99 ± 3.05% to 69.34 ± 4.11%; Figure 8A) as was the case under p38 MAPK inhibition (59.20 ± 4.70% to 55.15 ± 4.41%; Figure 8B). However, LA-induced hemolysis was significantly reduced from 55.23 ± 3.07% to 44.52 ± 4.45% upon CK1α blockade, as seen in Figure 8C. Finally, the addition of EPO showed no significant change in hemolysis (67.22 ± 2.99% to 60.15 ± 9.65%; Figure 8D).

Taken together, these observations seem to indicate that LA-induced cell death may be mediated through CK1α stimulation.

### 3.7. LA Elevates the Neutrophil-Lymphocyte Ratio (NLR)

**Figure 9:**
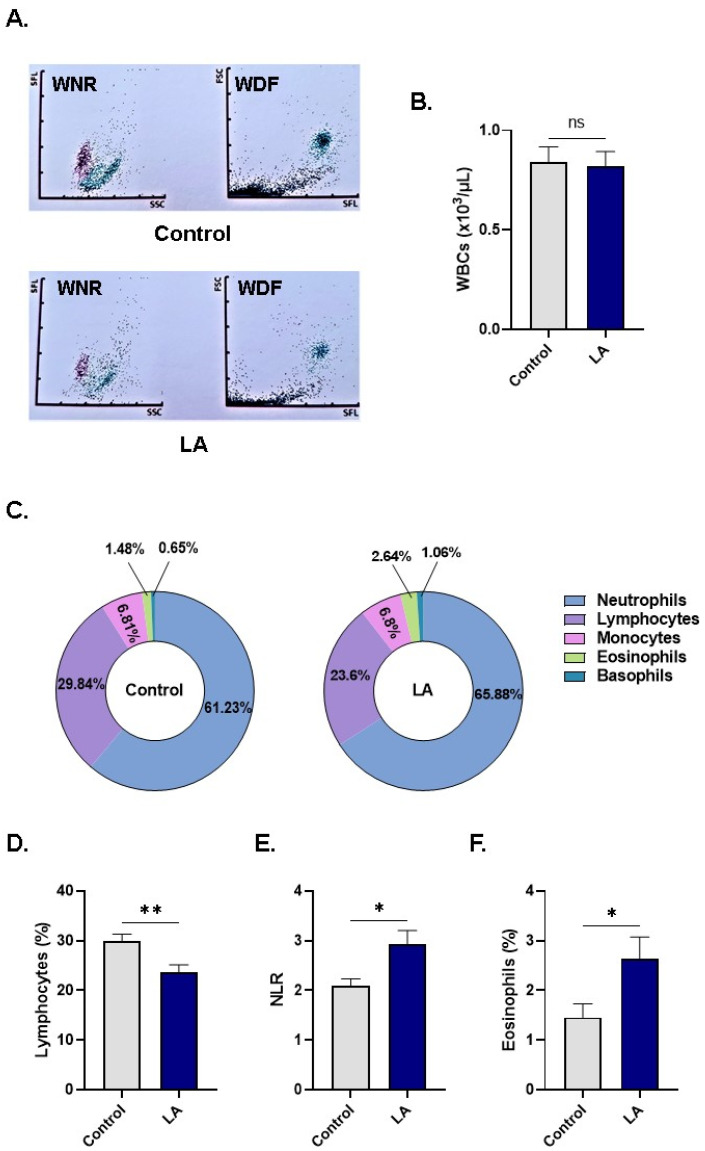
LA elevates NLR. (**A**) Representative dot plot scattergrams of control LA-treated peripheral WB cells. WNR channels show the distribution of WBCs in terms of side fluorescence (SFL) and SSC, while the WDF channel relates FSC and SFL. (**B**) Total WBC viability. (**C**) Differential count of WBC subsets. (**D**) Percentage of lymphocytes. (**E**) NLR. (**F**) Percentage of eosinophils. ns indicates not significant whereas * (*p* < 0.05) and ** (*p* < 0.01) indicate significantly different values from control conditions.

Based on the cytotoxicity of LA to RBCs, we sought to determine the range of other peripheral blood cells susceptible to LA. To this end, WB was treated with the vehicle control or with 100 μM of LA and examined for indices of cellular subsets. Figure 9A depicts changes in scattergrams of white cell differential (WDF) and white cell nucleated (WNR) channels of control and LA-treated WB. While total WBC viability slightly and insignificantly decreased upon LA treatment (0.83 ± 0.07 × 10^3^ cells/μL to 0.81 ± 0.07 × 10^3^ cells/μL; Figure 9B), further analysis of WBC subsets in Figure 9C,D, however, revealed significantly diminished proportions of lymphocytes (29.84 ± 1.45% to 23.60 ± 1.55%).

In light of the reduced percentage of lymphocytes and the emerging role of NLR as a stress marker, it was then of interest to probe possible disturbances in NLR of LA-treated WB. Indeed, treatment with LA caused a significant elevation in NLR from 2.10 ± 0.13 to 2.93 ± 0.27, as seen in Figure 9E. Notably, the percentage of eosinophils was also significantly higher upon LA exposure (1.45 ± 0.27% to 2.64 ± 0.43%; Figure 9F). No significant changes were detected in RBC or platelet indices (Figures S2 and S3, respectively).

Collectively, these observations reveal that LA is selectively toxic to lymphocytes which disturbs peripheral WBC proportions reflective of significant systemic stress.

## 4. Discussion

CVD remains a leading cause of death worldwide, for which hypertriglyceridemia is a recognized risk factor [53]. In this report, we show that LA causes Ca^2+^-dependent premature RBC death associated with elevated ESR, dysregulated ionic homeostasis, cell shrinkage and granularity, oxidative injury and accumulation of peroxides, and stimulation of CK1α. These outcomes demonstrate the importance of LA and its pathophysiological mechanisms, particularly in terms of its potential impact on the risk of CVD.

Despite the scarcity of data regarding blood levels of LA, in one small study, values of up to 37 μM were detected in apparently healthy participants [54]. However, given the inherently wide variation in dietary habits, fluctuations in circulating blood levels of LA are expected to be not uncommon, with higher concentrations that parallel typical hypertriglyceridemia expected to be reached in CVD. The range used in our study (50–250 μM) is thus expected to be encountered in vivo in both healthy and diseased individuals and is in line with concentrations previously used in vitro [55,56,57].

Excessive hemolysis (Figure 1) may lead to anemia [58], and circulating naked hemoglobin undergoes auto-oxidation or oxidation by other agents, which precipitates inflammatory conditions. Of note, decreased nitric oxide levels compromise endothelial elasticity and foster atherogenic sequelae [41], whereas oxidation of LDL-C particles by the heme ring similarly contributes to plaque formation [59], further arguing for the detrimental role of hemolysis in CVD.

An independent risk factor for cardiovascular complications, anemia increases the risk of death by more than two times among patients with kidney disease and CVD compared to those without anemia [60] and has also been identified as a risk factor for acute coronary syndrome [61] and ischemic heart disease [62]. Chronic anemia can lead to cardiac enlargement and left ventricular hypertrophy as a result of volume overload [63]. While this suggests a potential link between LA-induced hemolysis and CVD, further, long-term studies on animal models are required to assess the contribution of LA to the development of chronic anemia.

Our results also introduce a novel role of LA in human RBCs, which is the stimulation of eryptosis (Figure 2). Under physiological conditions, eryptosis serves as a mechanism by which senescent and injured cells are removed from the circulation by macrophages, as the display of PS on the RBC surface facilitates phagocyte binding and engulfment [39]. However, inordinate eryptosis results in accelerated disposal of cells and may thus lead to anemia among other conditions [64,65] as alarmingly seen in heart failure [52,66].

Due to severely disrupted structural integrity, eryptotic cells exhibit a significantly increased propensity to adhere to endothelial wall, promoting blood stasis, and adversely affecting blood rheology [47,67]. This manifestation is highlighted by the increased ESR observed in LA-treated cells (Figure 2D), indicating high blood viscosity. A few studies have reported associations between ESR and CVD [68,69,70]. For example, ESR was identified as a significant predictor of heart failure in a study of middle-aged men [68] and a long-term independent predictor of coronary heart disease among men and women [69]. It is, therefore, plausible to speculate that LA may participate in the development or complications of CVD by stimulation of eryptosis.

Calcium regulates essential cellular processes, most notably death and survival. We found that the eryptotic potential of LA is mediated through Ca^2+^ influx (Figure 3), possibly implicating uncontrolled Ca^2+^ channel activity in PS externalization (Figure 2E–G). Scramblases, flippases, and floppases are enzymes that control the movement of phospholipids, most notably PS, within the plasma membrane, and require Ca^2+^ as a cofactor for activity [71]. Increased entry of Ca^2+^ and subsequent disturbance of these Ca^2+^-dependent enzymes culminates in PS externalization and overt eryptosis.

Many channels and transporters are also sensitive to fluctuations in cytosolic Ca^2+^ levels, among which K^+^ channels are the most notable [72]. When Ca^2+^ activity increases, the opening of Ca^2+^-responsive K^+^ channels forces the escape of KCl and water, which explains the loss of cellular volume observed in our study (Figure 4). It is important to note that a direct interaction of LA with aquaporins and subsequent loss of water cannot be ruled out, considering that cell shrinkage did not apparently require the presence of Ca^2+^ (Figure 4C). This is in contrast to LA-induced acanthocytosis (Figure 4F,G), which was significantly abrogated upon the elimination of Ca^2+^ (Figure 4F,H). Acanthocytes are susceptible to splenic digestion [73], again increasing the risk for hemolytic anemia [74]. Notably, membrane blebbing and surface granularity reflect increased stiffness which may be ascribed to cytoskeletal damage and oxidative injury, which, in turn, was also demonstrated to ensue following Ca^2+^ influx (Figure 5 and Figure 7).

Oxidative stress and lipid peroxidation contribute to atherosclerosis by oxidation of LDL-C particles that accumulate in the subendothelium and trigger an inflammatory immune response and plaque formation [34,35,36]. Not only that, but oxidative stress may also contribute to the other mechanisms investigated in the current study, including hemolysis [75,76] and eryptosis [64], and can be assessed by ESR [67]. Counteracting oxidative stress may thus be effective in reducing associated inflammation and damage, and can include dietary interventions with naturally-occurring antioxidants [77]. These free-radical scavengers inhibit or delay the oxidative damage of molecules by accepting or donating electrons to convert the radical to nontoxic species [78,79]. They can also control the activity of ROS-generating enzymes or increase that of antioxidant enzymes, as observed in the current study [80]. The antioxidant defense system includes antioxidants that are enzymatic, like CAT and SOD (Figure 5), and non-enzymatic like GSH (Figure 6); the most prevalent naturally-occurring antioxidant [81], vitamins E and C, β-carotene, and urate [82].

Animal studies [83,84], systematic reviews and meta-analyses of prospective cohort studies [85], and randomized controlled trials [86] have suggested that foods and beverages rich in antioxidants could play a preventive role in modifying the risk of CVD through counterbalancing the effects of ROS and nitrogen species involved in the atherosclerotic process [87]. For example, randomized controlled trials [88,89] showed that diets enriched with cysteine and glycine, the precursors of GSH, fully restore cellular glutathione synthesis and reduce cell damage caused by direct oxidizing effects on proteins and lipids (Figure 7), and on DNA, thus serving as the first cellular defense line against ROS. Further, antioxidant enzymes that glutathione uses as cofactors, serve as the second defense line [90]. Concurrently, decreased GSH synthesis (Figure 6) has been shown to precede oxidative stress and may contribute to atherogenesis [91,92].

Our results also seem to point to a potential role for CK1α in LA-induced RBC death (Figure 8C), which is in congruence with the established participation of the enzyme in the toxicity of a wide array of xenobiotics as we have previously reported [39,42,91,92]. To the best of our knowledge, this is the first report to identify CK1α as a molecular target for LA. Thus, blocking the activity of this enzyme may, at least in theory, ameliorate LA-induced RBC death.

In conclusion, this study presents novel mechanistic insights into the nature of interactions between LA and erythrocytes with important implications for CVD risk and development. Importantly, the eryptotic properties of LA and associated underlying mechanisms are orchestrated by Ca^2+^ influx through disrupted ion channel activity. These observations provide a working platform for a more thorough understanding of the pathophysiology of individual FAs and, thus, the optimization of dietary interventions for CVD prevention and management.

## Data Availability

The data that support the findings of this study are available from the corresponding author, M.A.A., upon reasonable request.

## Supplementary Materials

The following are available online at https://www.mdpi.com/article/10.3390/cells10123388/s1, Figure S1: Dose-dependent hemolytic activity of IMC. Figure S2: RBC indices. Figure S3: Platelet indices.
