# Supplementary material for: Lauric Acid, a Dietary Saturated Medium-Chain Fatty Acid, Elicits Calcium-Dependent Eryptosis
Source: Cells. 2021 Dec 1;10(12):3388. doi: 10.3390/cells10123388 (PMC8699421; doi:10.3390/cells10123388)
Supplement: Supplementary file 1 [file cells-10-03388-s001.zip › cells-1436975-supplementary.pdf]

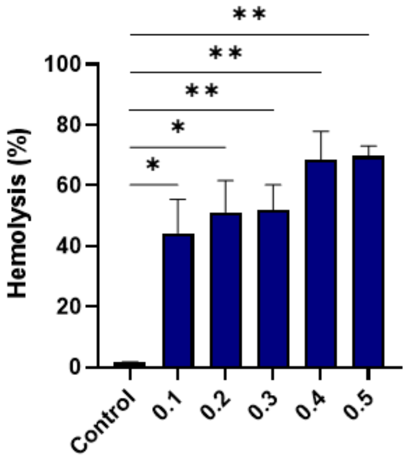

Figure S1. Dose-dependent hemolytic activity of IMC. RBCs were treated with the vehicle or with 0.1-0.5 μM of IMC for 24 h at 37 ^{o}C and hemolysis was then quantified.

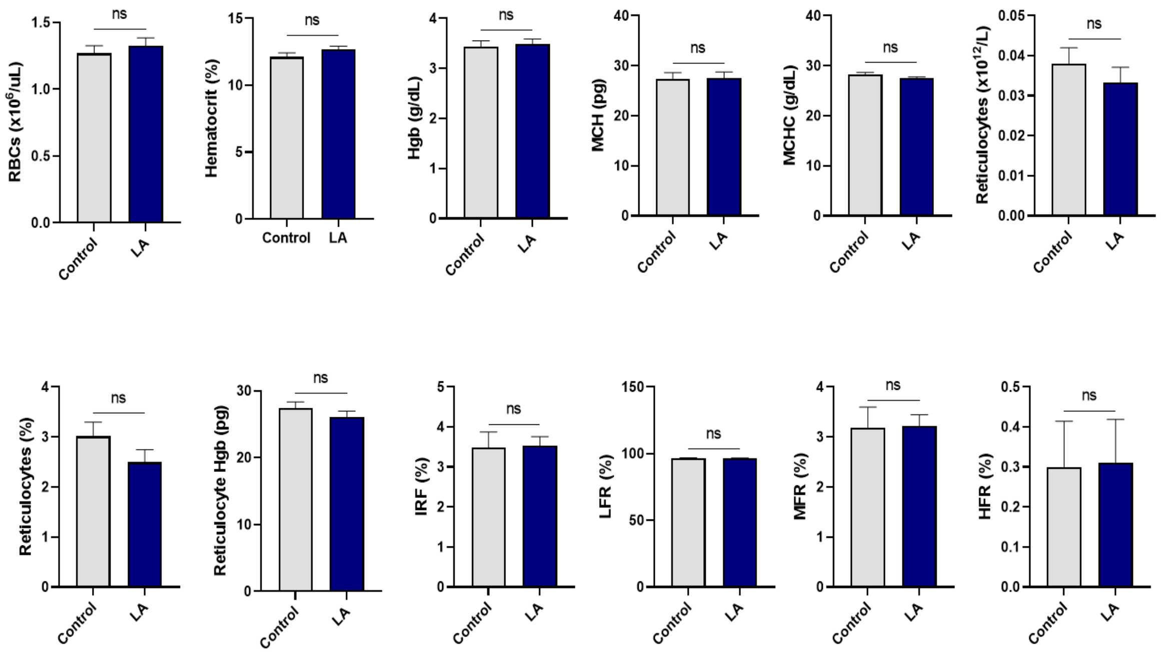

Figure S2. RBC indices. WB was treated with the vehicle or with 100 μM of LA for 24 h at 37C and RBC indices were analyzed. (**A**) RBC viability. (**B**) Hematocirt. (**C**) Hgb. (**D**) MCH. (**E**) MCHC. (**F**) Reticulocyte viability. (**G**) Reticulocyte percentage. (**H**) Reticulocyte Hgb. (**I**) IRF. (**J**) LFR. (**K**) MFR. (**L**) HFR.

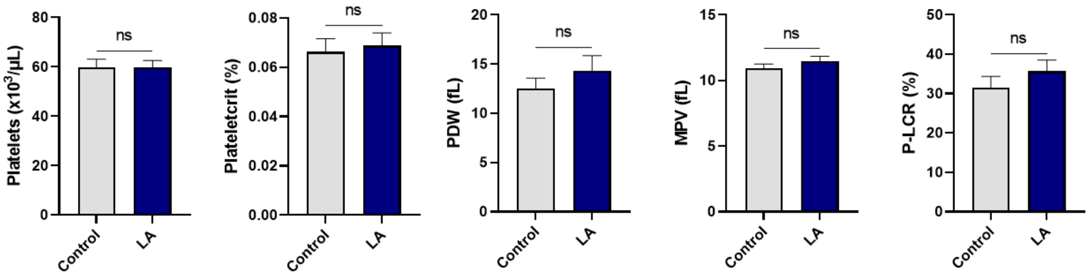

Figure S3. Platelet indices. WB was treated with the vehicle or with 100 μM of LA for 24 h at 37C and platelet indices were analyzed. (**A**) Platelet viability. (**B**) Plateletcrit. (**C**) PDW. (**D**) MPV. (**E**) P-LCR.
